# Aldehyde dehydrogenase 2 rs671 polymorphism and multiple diseases: protocol for a quantitative umbrella review of meta-analyses

**DOI:** 10.1186/s13643-022-02050-y

**Published:** 2022-09-02

**Authors:** Zhengting He, Qi Guo, Yikai Ling, Chuan Hong, Yuqing Liu, Xurui Jin, Porama Thanaporn, Duan Zhao, Leiting Wang, Liang Liu, Lijing L. Yan

**Affiliations:** 1 Global Health Research Center, Duke Kunshan University, No. 8 Duke Avenue, Kunshan, Jiangsu 215316 China; 2 Department of Epidemiology, Bloomberg School of Public Health, Johns Hopkins University, 615 North Wolfe Street, Baltimore, MD 21205 USA; 3 Duke Kunshan University, No. 8 Duke Avenue, Kunshan, Jiangsu 215316 China; 4 Department of Biostatistics & Bioinformatics, Duke University, 2424 Erwin Road, Durham, NC 27705 USA; 5 MindRank AI Ltd., Hangzhou, Zhejiang 310000 China; 6 Department of Internal Medicine, University of Michigan Medical School, 1301 Catherine St, Ann Arbor, MI 48109 USA; 7 School of Public Health, Hong Kong University, 7 Sassoon Road, Pokfulam, Hong Kong; 8 Duke Global Health Institute, Duke University, 310 Trent Drive, Durham, NC 27710 USA; 9 School of Public Health, Wuhan University, No. 115 Donghu Road, Wuhan, Hubei 430071 China; 10 Institute for Global Health and Development, Peking University, No. 5 Yiheyuan Road, Beijing, 100871 China

**Keywords:** ALDH2, rs671, Polymorphism, Umbrella review, Meta-analysis

## Abstract

**Background:**

The mutant allele (*2) of aldehyde dehydrogenase type 2 (ALDH2) caused by a single nucleotide variant (rs671) inhibits enzymatic activity and is associated with multiple diseases. In recent years, an explosive number of original studies and meta-analyses have been conducted to examine the associations of ALDH2 rs671 polymorphism with diseases. Due to conflicting results, the overall associations of ALDH2 rs671 polymorphism and multiple diseases remain unclear.

**Methods:**

A quantitative umbrella review will be conducted on meta-analyses of genetic association studies to examine the pleiotropic effects of ALDH2 rs671, mainly including cardio-cerebral vascular disease, diabetes mellitus, cancer, neurodegenerative disease, and alcohol-induced medical disease. A search of relevant literature according to comprehensive search strategies will be performed on studies published before July 1st, 2022 in PubMed, MEDLINE Ovid, Embase, Cochrane Database of Systematic Reviews, and Web of Science. Study selection, data extraction, methodology quality assessment, and strength of evidence assessment will be conducted by two reviewers independently and in duplicate. Included meta-analyses will be grouped by outcomes. Data conflicts and overlap between meta-analyses will be managed through updated standardized and customized methods including the calculation of CCA for study selection reference, application of Doi plots to assess small-study effects and others. Evidence from included meta-analyses will be quantitatively synthesized by overlap-corrected analyses and meta-analysis using primary studies.

**Discussion:**

This umbrella review is expected to generate systematic evidence on the association between ALDH2 rs671 and diseases. Specific approaches were developed to address key challenges in conducting an umbrella review, including assessment tools of methodology and evidence quality of meta-analyses, methods to manage overlap between meta-analyses, a “stop-light” plot to summarize key findings. These approaches provide applicable methods for future umbrella reviews of meta-analyses on genetic association studies.

**Trial registration:**

CRD42021223812

**Supplementary Information:**

The online version contains supplementary material available at 10.1186/s13643-022-02050-y.

## Background

Mitochondrial aldehyde dehydrogenase (aldehyde dehydrogenase type 2, ALDH2) belongs to the aldehyde dehydrogenase superfamily of proteins, which shows the highest affinity for acetaldehyde among enzymes oxidizing aldehydes [1, 2]. In the alcohol metabolism pathway, alcohol dehydrogenase oxidizes ethanol to acetaldehyde, and then ALDH2 catalyzed the oxidation of acetaldehyde to acetate, which is excreted to the blood and finally converted to CO_2_ [3]. Besides ethanol metabolism, ALDH2 is a key enzyme involved in the degradation of toxic reactive acetaldehydes, such as 4-hydroxy-2-nonenal (4-HNE) and malondialdehyde (MDA), into nontoxic acetic acid [4]. Apart from liver, ALDH2 is also expressed in multiple tissues that require high mitochondrial content, such as the heart, kidney, lung, and brain [5].

A common ALDH2 deficient allele (ALDH2*2, Glu504Lys) is caused by a single nucleotide polymorphism (SNP), which is a G to A mutation at codon 504 in exon 12 of ALDH2 gene located on chromosome 12q24 (rs671 G>A), resulting in the substitution of glutamate (Glu) with lysine (Lys) in subsequent translation process. Both heterozygous and homozygous ALDH2*2 carriers (ALDH2*1/*2 or ALDH2*2/*2) have enzymatically inactive ALDH2 [1, 6]. ALDH2*2 is largely limited to East Asian populations, affecting approximately 40% of East Asian populations (560 million) and 8% of global populations [7].

ALDH2 deficiency is a double-edged “sword”. Lacking functional ALDH2 enzyme causes rapid accumulation of acetaldehyde, resulting in facial flushing reactions in many east Asians [7]. On the one hand, flushing and dysphoria urge the carriers to drink less, reducing the risks of initiation and progression of alcohol-related diseases. On the other hand, local culture and social norms in certain regions still promote drinking despite this reaction, resulting in adverse health effects.

ALDH2 is activated by protein kinase C isotype–*ε*, and functions as a protector against oxidative stress and apoptosis [8]. Enzymatically inactivated ALDH2 causes accumulation of reactive aldehydes, generating reactive oxygen species, upregulating downstream enzymes involved in stress-response pathways activation, such as P38 mitogen-activated protein kinase [9] and AMP-dependent protein kinase [10], resulting in ischemic cardiomyopathy [10, 11], aberrant adipogenesis [12], and damage on nervous system [13, 14]. End products of non-oxidized acetaldehyde, such as 4-HNE and MDA, can easily diffuse through cell membranes, and may indirectly impact the nuclear genome via the formation of DNA and protein adducts, activating apoptosis pathways and driving oxidative stress-induced cell apoptosis [15, 16]. These mechanisms lead to pleiotropic associations of ALDH2 loss of function mutation with multiple disorders, including cardiovascular diseases, diabetes mellitus, neurodegenerative diseases, alcohol-induced pathophysiology, upper aerodigestive tract cancer, and pain, etc [17].

An explosive number of meta-analyses have been conducted on the associations of ALDH2 rs671 polymorphism with diseases, with increasing publication frequencies over the years and conflicting results [18–29]. Typically, these meta-analyses focused on single disease outcome such as hypertension, cardiovascular disease, or cancer, and showed both deleterious and protective associations of ALDH2 rs671 polymorphism with these diseases. These studies warrant synthesis of evidence to explore reasons for conflicts between results and to provide clarity to healthcare decision-makers and people affected by the deficiency of ALDH2.

Umbrella review (also known as overview of systematic reviews), is a publication type, emerging as the result of explosive growth of systematic reviews. It allows findings of separate reviews to be compared and contrasted, formulating a comprehensive but concise conclusion, providing decision-makers with the evidence they need [30]. In terms of the hierarchy of evidence synthesis methods, some consider umbrella reviews to be in the highest evidence level, superseding meta-analysis, systematic reviews, and individual studies [31]. The objective of this paper is to examine the pleiotropic associations between ALDH2 rs671 polymorphism with multiple disease outcomes, by conducting an umbrella review to quantitatively synthesize evidence from published meta-analyses of genetic association studies.

## Methods

Our umbrella review will adhere to the PRISMA 2020 statement: an updated guideline for reporting systematic reviews [32]. Preferred Reporting Items for Systematic Reviews and Meta-analysis Protocols (PRISMA-P) checklist [33] was adopted to guide the development of this protocol. Our review protocol was registered with the International Prospective Register of Systematic Reviews (PROSPERO) (registration number CRD42021223812). Results of this umbrella review will be published in a peer-reviewed journal. Potential changes to the protocol will be described in the final umbrella review report.

In the following sections, we will refer to an umbrella review following Overviews of Reviews guidelines in the Cochrane Handbook for Systematic Reviews of Interventions [34] as a “Cochrane overview” [34]. Searches, study selection, data extraction, methodology quality assessment, and strength of evidence assessment will be conducted by two reviewers independently and in duplicate, with disagreements solved through discussion with a third reviewer.

### Study selection

#### Study designs

A systematic review is defined as a review with clearly stated objectives, reported search strategy and sources searched, eligibility criteria, study selection process, and reproducible methods to identify, extract, and synthesize the findings of included primary studies.

Systematic reviews utilizing quantitative synthesis methods or mixed synthesis methods (both qualitative and quantitative methods) on evidence are eligible for inclusion. If a systematic review evaluated multiple genetic variants, it is eligible for inclusion if at least part of the data synthesis was performed on the association of ALDH2 rs671 polymorphism and disease. To be eligible for inclusion, the types of primary studies included in the review should be cohort, nested case-control, or case-control studies.

#### Population

Meta-analyses with case and control groups will be included. Cases are defined as patients with cardio-cerebral vascular disease, diabetes mellitus, cancer, neurodegenerative disease, or alcohol-induced medical disease, diagnosed by symptoms, physical examinations, or radiological examinations by qualified physicians, according to criteria suggested by clinical guidelines. Patients with self-reported diseases will be excluded. Controls are defined as healthy individuals, whose sources can be population-based or hospital-based.

#### Exposure

Meta-analyses examining ALDH2 rs671 polymorphism, genotyped by polymerase chain reaction, DNA microarrays, DNA sequencing, or other molecular biological techniques, will be included.

#### Comparator

The comparator is ALDH2 major allele, detected by methods above.

#### Outcomes

Outcomes of interest are susceptibility to the following diseases, which were formed through consultation of published narrative reviews [17] and preliminary searches.

1. Cardio-cerebral vascular disease:
  1. Essential hypertension
  2. Coronary artery disease and myocardial infarction
  3. Ischemic stroke
2. Diabetic mellitus and diabetic retinopathy
3. Cancer:
  1. Esophageal cancer
  2. Gastric cancer
  3. Hepatocellular carcinoma
  4. Pancreatic cancer
  5. Colorectal cancer
  6. Head and neck cancer
  7. Breast cancer
4. Neurodegenerative disease:
  1. Alzheimer’s disease
  2. Parkinson’s disease
5. Alcohol-induced medical disease:
  1. Alcoholic liver cirrhosis
  2. Alcoholic pancreatitis

#### Language and publication status

Studies reported in English and published in peer-review journals are eligible for inclusion.

Through preliminary searches, we noticed discrepancies in the inclusion criteria regarding Hardy-Weinberg Equilibrium (HWE). Some meta-analyses excluded primary studies whose control groups departed from HWE [28], while others included these studies with or without sensitivity analysis in further statistical calculations [27, 29]. Currently, there are three procedures to address studies that depart from HWE in meta-analysis: (i) perform a sensitivity analysis by excluding studies that depart from HWE and studies without sufficient information to test for HWE; (ii) exclude studies with significant (*P* < 0.05, adjusting for multiple testing) deviation from HWE completely from meta-analysis; (iii) correct pooled OR and its variance to account for departure from HWE; of them, none of the procedures is clearly superior but procedure (i) is routinely adopted [35]. Thus, we will include meta-analysis that did not exclude primary studies whose control groups departed from HWE in the study selection stage, flag these studies and conduct sensitivity analyses.

A summary of the main study inclusion criteria is provided in Table 1.

**Table 1:** Study inclusion criteria

Items	Inclusion criteria
Language	1 Reported in English
Publication status	2 Published in peer-review journals
Exposure	3 ALDH2 rs671 polymorphism
Outcome	4 Cardio-cerebral vascular diseases, diabetes mellitus, cancer, neurodegenerative diseases, or alcohol-induced medical diseases
Study design	5 The study design was a systematic review 6 Evidence was synthesized utilizing quantitative methods 7 The study designs of primary studies were cohort, nested case-control, or case-control studies

### Data source

The search strategy was developed based on reference to several meta-analyses on ALDH2 polymorphism and diseases found during preliminary searches [18–29]. Three groups of keywords were used to conduct the search: “ALDH2” group, “polymorphism” group, and “systematic review” group. Boolean rule “AND” was used between each group, while Boolean rule “OR” was used within each group. The “ALDH2” group included: ALDH2, ALDH-2, “ALDH 2”, “aldehyde dehydrogenase type 2”, “aldehyde dehydrogenase 2”, “aldehyde dehydrogenase-2”, “aldehyde dehydrogenase II”, “mitochondrial aldehyde dehydrogenase”, ALDM, “alcohol metabolism”. The “polymorphism” group included: polymorphism*, variation*, variant*, mutation*, genotype*, allele*, “single nucleotide polymorphism”, SNP, rs671, G487A, 487Lys, 504Lys, “ALDH2*2”. The “systematic review” group adopted the “Literature reviews and meta-analyses search filter for Ovid Medline” developed by the Quebec chapter of Canadian Health Libraries Association (ASTED 3S) [36], and was modified to fit with searching on each database. (Detailed search strategy in each database is outlined in Additional file 1.)

Searches will be conducted using MeSH terms combining with title/abstract keywords for studies published prior to July 1st, 2022 on five databases: PubMed, MEDLINE (Ovid interface), Embase, Cochrane Database of Systematic Reviews, and Web of Science. The literature search will be limited to English. Citation abstracts and full text of search results will be imported to EndNote software. After excluding duplicates, they will be screened based on study selection criteria. Systematic searches in the databases will be supplemented by the “snowballing” strategy, i.e., reference lists of retrieved full-text papers will be screened to avoid potential omissions. Prior to quantitative evidence synthesis, searches will be updated to the latest availability.

**Fig. 1:**
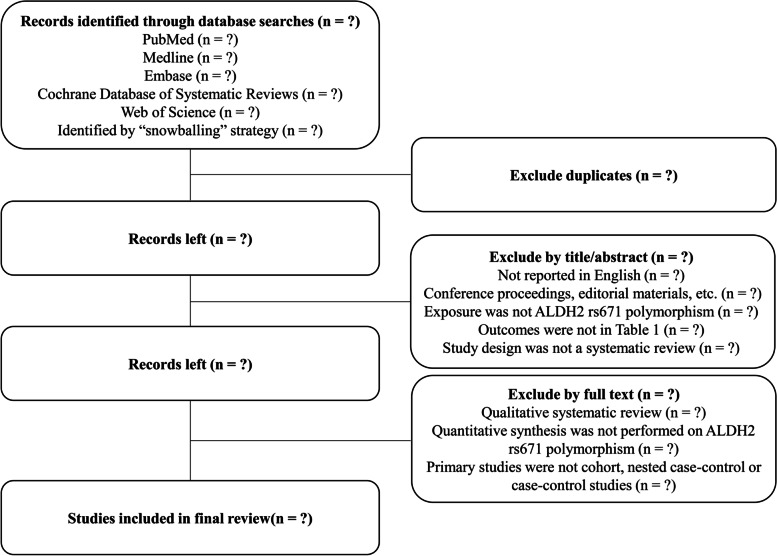
Flow diagram of the study selection process

Titles and abstracts of search records will be scanned against criteria 1 to 5 in Table 1. Full texts of studies meeting these criteria will be further scanned against criteria 6 to 7 in Table 1. A flow diagram of the study selection process is outlined in Fig. 1.

### Data extraction

The following information at meta-analyses level (2-1 in Additional file 2) will be extracted: name of the first author, year of publication, exposure of interest, comparison, outcome of interest, total number of participants, number of primary studies, study design of primary studies, length of follow-up of primary studies (if applicable), quality assessment method for primary studies, pooled effect size (ES), confidence interval (CI) or standard error (SE), measurement of ES, meta-analyses method, *p* value of pooled ES, genetic model, and heterogeneity (Cochrane *Q* or *I*^2^ statistics). The following information at primary studies level (2-2 in Additional file 2) will be extracted in each meta-analysis: name of the first author, year of publication, ethnicity, study design, source of control, alcohol consumption status, sex, exposure of interest, comparison, precalculated ES, CI or SE, measurement of ES, genetic model, *p* value for HWE *χ*^2^ test.

### Methodological quality assessment

The measurement tool to Assess the Methodological Quality of Systematic Reviews (AMSTAR) [37] is widely used by umbrella reviewers of quantitative systematic reviews. However, there are challenges for applying AMSTAR to rate. For instance, questions within the AMSTAR checklist are often multi-faceted, which complicates the rating process and implicates more subjective factors in the assessment [38]. In 2017, original developers of AMSTAR developed an updated measurement approach called a critical appraisal tool for systematic reviews that include randomized or non-randomized studies of healthcare interventions, or both (AMSTAR2), adapting to a more detailed assessment of systematic reviews with moves to extend AMSTAR to incorporate systematic reviews of observational studies [39]. Good reliability for most items on sample systematic reviews was reported [39]. Currently, no consensus has been made on which approach is clearly superior, but an ongoing study is being performed to assess these approaches [40].

AMSTAR2 is a 16-item checklist with 7 critical items, mainly for quality assessment of systematic reviews of health interventions [39]. It comprehensively evaluates the potential bias in search, study selection, data extraction, data presentation, risk assessment, statistical methods, and funding sources. For each item, the answer will be given as “yes”, “partial yes”, “no”, or “inapplicable”. It is not appropriate to generate a quality score when using AMSTAR/AMSTAR2 [38, 39]. Instead, rating overall confidence of each review will be graded as “high”, “moderate”, “low”, or “critically low” according to the number of critical flaws or non-critical weaknesses in these items [39]. To fit AMSTAR2 with meta-analyses of observational studies, we modified several items in AMSTAR2 checklist with the reference of AMSTAR [37], ROBIS [41], and quality assessment approaches for observational studies, such as Newcastle-Ottawa Scale (NOS) [42] and Strengthening the Reporting of Genetic Association studies (STREGA) [43]. (Modified AMSTAR2 and quality assessment table are in Additional file 3.)

No re-assessment on primary studies will be conducted. We will adopt the quality assessment approach and results performed by included meta-analyses’ authors.

### Strength of evidence assessment

Grading of Recommendations Assessment, Development and Evaluation (GRADE) [44] is mainly adopted by umbrella review authors for assessing systematic reviews using quantitative synthesis methods. However, gaps remain in application of GRADE in systematic reviews [45]. For instance, the subjectivity associated with the application of each criterion of GRADE has been reported [38, 46]. Even among most experienced reviewers, reaching agreements on the overall strength of evidence can be difficult [46]. Efforts have been made to address these gaps, including proposal of a more algorithmic approach to judge quality of evidence based on GRADE [38, 47], clarification of factors considered in applying each criterion of GRADE [48], and development of Evaluating Strength of the Quantitative Evidence at the Level of an Umbrella Review [49].

Since the exposure evaluated is a SNP, nearly all included meta-analyses are based on evidence from observational studies. Consequently, applying GRADE will inherently start with a level of “low quality” [50]. This may result in a poor reflection of the evidence quality variations between reviews, since the quality of evidence of most reviews will be limited to “low” or “very low”. Meanwhile, since all included studies are meta-analyses, which are methodologically unified, utilizing statistical results, such as *p* value of pooled ES or range of CI, may result in a clear appraisal. Therefore, we decided to adopt the method for grading evidence of meta-analyses in several recent published umbrella reviews [51–54], which use the *p* value of *Z*-test on pooled ES as the main criterion for grading evidence (Additional file 4).

No re-assessment on primary studies will be conducted.

### Data preparation

Before data synthesis, included meta-analyses will be grouped based on outcomes of interest. Meta-analyses reporting multiple outcomes will be categorized in the respective outcome group, deconstructed and evaluated separately, only on the outcomes of interest in the respective group.

For each meta-analysis, if precalculated ES for primary studies were not fully presented, or if the ES of primary studies for some of the genetic models were not calculated while there is sufficient individual participant data (number of cases and controls under each genotype) to conduct the calculation, individual participant data will be used to supplement the calculation. For each primary study without reporting whether the control group followed HWE, *χ*^2^ test (significant level alpha will be set at 0.05 level) will be used to measure whether the control group followed HWE. The results will be filled in the data extraction form (2-2 in Additional file 2).

Through preliminary searches, we discovered a substantial number of conflicts between the precalculated ES and CI on the same primary studies included in multiple meta-analyses. This may originate from errors in calculation or data extraction of included meta-analyses. Conflicts will be resolved according to the following pre-specified procedure: (i) first, ES will be recalculated using individual participant data to prevent calculation errors in each meta-analysis. (ii) If the error is caused by data extraction, or if there is no sufficient individual participant data presented in the meta-analyses, the primary study will be reviewed and data will be extracted. (2-3 in Additional file 2) The finalized individual participant data will be decided by comparison with the data extracted by each meta-analysis, and further discussions between reviewers. ES and CI will subsequently be calculated based on the finalized individual participant data. (iii) If there is no sufficient data presented in the primary study to calculate ES, the precalculated ES in the meta-analyses with the highest methodology quality will be adopted.

### Managing overlap

Managing overlap between systematic reviews is significant in conducting umbrella reviews. Pieper et al.’s review of 60 umbrella reviews showed a substantial amount of duplicate primary studies in included reviews; however, only half of the authors addressed the overlap, while the rest disregarded this issue [55]. This may lead to underestimation of the overlap, since primary studies included in multiple reviews are calculated multiple times, resulting in disproportionate statistical power [55]. On the other hand, researchers need to be cautious of overestimation of overlap, when multiple reviews include the same primary study, but non-overlapping data is extracted from this study [55].

Based on Pieper et al.’s method, a citation matrix will first be presented, and the covered area (CA) and corrected covered area (CCA) will be calculated as measures of degree of overlap for each health outcome with multiple reviews included [55]. (Format of citation matrix and formula for CA and CCA are in Additional file 5)

In dealing with duplicate primary studies in quantitative umbrella reviews, early published studies chose to decrease the degree of overlap by removing part of the included meta-analyses based on completeness, recency, and methodological quality, which introduces bias of its own [56, 57]. In 2013, Munder et al. [58] reported a statistical method to deal with duplicate primary studies in their meta-meta-analysis and was further adopted by two other meta-meta-analyses [59, 60], which requires transforming ES to Fisher’s *Z* and calculating overlap-adjusted weight for each meta-analysis. (Detailed formulas and explanations are in Additional file 6) Preliminary searches indicated that most included meta-analyses used odds ratio as the measurement of the ES. Moreover, the method above did not consider the sample size of each primary study, which is highly related to the standard error of odds ratio, which determines the overlap-adjusted weight, of each meta-analysis. Thus, transforming the ES to Fisher’s *Z* and adopting the method above is not meaningful. To achieve a smaller overlap without removing too many primary studies, the corrected covered area (CCA) will be calculated for each disease after dropping one or more meta-analyses; the combination with the smallest CCA will be selected for further analyses if it drops the least number of primary studies compared with other combinations with similar CCA. When two meta-analyses include identical primary studies or one’s is completely a subset of another for certain outcomes, only the latest meta-analyses would be included. If the overlap management methods lead to only one meta-analysis for certain outcomes, the results of that meta-analysis would be directly used for those outcomes. For meta-analyses that include similar primary studies but focus on different features/aspects/subgroup analyses and others, we would still include them to ensure there is a sufficient number of meta-analyses to conduct the umbrella review on each of those different features/aspects/subgroup analyses [61].

According to Cochrane Handbook for Overviews, when it is possible for reviewers to avoid double-counting outcome data from overlapping reviews by ensuring that each primary study’s data is extracted only once, reviewers should include all relevant reviews and follow this approach, though it is time-intensive and methodologically complex [34]. We define the criteria for correcting overlap in quantitative synthesis as ensuring all primary studies in all relevant meta-analyses are included, and ensuring data from each primary study is extracted only once.

### Quantitative synthesis

Calculations will be conducted based on available data assuming the following genetic models: allelic (A vs. G), dominant (AG + AA vs. GG), heterozygous (AG vs. GG), homozygous (AA vs. GG), and recessive (AA vs. GG + AG).

#### Overlap-corrected analysis

For each outcome, if more than one meta-analysis is included, overlap between meta-analyses will be corrected based on a formed procedure. If a certain meta-analysis which included all primary studies that were included in other meta-analyses existed, and if these meta-analyses extracted identical data from these primary studies, which is considered as a “complete overlapping” meta-analysis, pooled ES and CI of this meta-analysis will be adopted as overlap-corrected results. Otherwise, precalculated ES and CI of all primary studies being included in all meta-analyses will be used to conduct a meta-analysis under each genetic model, ensuring that each primary study is counted only once. As described in the previous section, similar studies with different features/aspects/subgroup analyses and others will be kept if previous overlap managing steps leave too few studies for meta-analyses. Precalculated ES and CI will be converted to Log Odds Ratio (Log_OR) and SE of Log_OR (formula is in Additional file 7). Heterogeneity and inconsistency across meta-analyses will be investigated and measured using Cochran’s *Q* test [62] and *I*^2^ test [63] respectively. A random effects model (Hartung-Knapp-Sidik-Jonkman adjustment method) [64] will be adopted for all outcomes to account for heterogeneity between studies and allow the effect estimates generalizing to target populations. Sensitivity analysis will be performed on primary studies whose control group deviate from HWE, or information of whether the control group followed HWE or not is unavailable. Funnel plots [65], Egger’s tests [66], Doi Plot [67], and LFK index [67] will be performed to assess the small-study effects of included primary studies. To assess the possible presence of small-study effects, sensitivity analysis will be performed by sequentially dropping one inter-study at a time to detect the impact of each inter-study on pooled ES. Subgroup analysis will be performed to explore possible sources of heterogeneity, and will be conducted based on available subgroup individual participant data or precalculated ES of primary studies on meta-analyses level. Primary studies will be stratified by ethnicity, gender, alcohol consumption, and source of control under each genetic model. Strength of each finding will be graded based on the “Strength of evidence assessment” procedure. A cumulative meta-analysis, ranked by year, will be performed to detect small-study effects.

#### Meta-analysis using primary studies

Subsequently, primary studies included by the eligible meta-analyses will be identified and used as a unit of analysis to perform an extensive meta-analysis and provide estimates on ALDH2’s ALDH2*2 allele’s effect towards multiple diseases. All primary studies will be included only once to prevent overlap.

Assessment of risk of bias will also be applied. Funnel plots [65], Egger’s tests [66], Doi plot and LFK index [67] will be performed to assess the small-study effects of included primary studies.

For each outcome, pooled ES and CI of included primary studies will be transformed to Log Odds Ratio (Log_OR) and SE of Log_OR. Heterogeneity across primary studies will be investigated and measured using Cochran’s *Q* test [62] and *I*^2^ test [63]. A random effects model (Hartung-Knapp-Sidik-Jonkman adjustment) [64] will be adopted for all outcomes.

#### Data presentation

Under each genetic model, to briefly summarize and present the results of included meta-analyses, pooled ES and CI will be presented as a “stop-light” plot [30] on the overall effects of ALDH2 rs671 polymorphism and diseases risks. Pooled ES and CI of all outcomes of interest calculated during the overlap-corrected analysis will be presented as a “stop-light” plot [30]. In the plot, outcomes which ALDH2 polymorphism has a higher disease risk will be filled with “red”, while the corresponding colors for a lower risk of disease and no effect (statistically insignificance) are “green” and “yellow”.

All statistical calculations will be conducted with R (R Foundation for Statistical Computing, Vienna, Austria, version 4.1.2), and R package “metafor” [68].

### Methodology summary

As a relatively new and emerging method for synthesizing evidence, gold standard guidance for conducting umbrella reviews is currently lacking, and challenges have been reported on umbrella review methods [45, 69]. We identified several challenges that have not been fully addressed in previous umbrella reviews, developed and refined several approaches, and will apply them in our umbrella review:

(i) Quality assessment: to fit quality assessment approaches for meta-analyses including observational studies, we specified and modified AMSTAR2 [39], and selected a checklist (Additional file 3), to assess methodological quality and grade strength of evidence.
(ii) Missing data and data conflicts: to supplement potential missing or not fully presented results in a certain meta-analysis, when there is sufficient individual participant data in the meta-analysis for us to conduct the calculation, we will supplement the calculation, which results will subsequently be adopted in quantitative synthesis. To solve data conflicts on a primary study between multiple meta-analyses, a sequential procedure was developed by recalculating odds ratio and CI using individual participant data, re-extracting data on primary study level, and deciding by quality assessment results.
(iii) Managing overlap: to report the degree of overlap between included meta-analyses, we will conduct citation matrices of primary studies and report CA, CCA [55]. For each disease, the combination with the smallest CCA will be selected for further analyses unless too few studies are left; under such condition, similar studies with different features will be included to ensure the feasibility of meta-analyses. To deal with overlap between meta-analyses and reduce bias of pooled ES, precalculated ES and CI of all primary studies included in all meta-analyses will be used to reconduct a meta-analysis as the overlap-corrected results, and data of each primary study will be ensured to be counted only once.
(iv) Summarizing key findings in a brief assessable format: “stop-light” plots [30] will be presented on results of all included meta-analyses, and on findings calculated during overlap-corrected analysis.

## Discussion

This umbrella review will systematically review the pleiotropic associations between ALDH2 rs671 polymorphism and multiple diseases. Understanding the associations between ALDH2 polymorphism and diseases will help decision-makers in frontline healthcare pay special attention and propose targeted prevention plans for populations with ALDH2 deficiency, constituting approximately 560 million populations. Synthesizing evidence from existing meta-analyses helps to resolve controversial results benefiting from a larger statistical power, providing a comprehensive and concise conclusion. Several challenges in conducting umbrella reviews have been addressed in our protocol, which will at least provide an applicable approach for future umbrella reviews of meta-analyses of genetic association studies for pertinent gene-disease associations.

We acknowledge several limitations in our study. Populations of primary studies stemming from a same dataset may have some extent of overlap. For instance, two primary studies included in different meta-analysis might be case-control studies utilizing the same case group. Since we will not extract data on primary studies level, it may not be possible to detect the overlapping populations in primary studies. But we anticipate these circumstances are relatively rare and the impact of which can be degraded by a large statistical power of all primary studies included. Population stratification cannot be addressed with single variant data; however, based on pilot studies, majority of the studies were conducted in east and south Asian populations; thus, we anticipate the effects of population stratification are small. Other limitations are that language of included studies is limited to English, which might lead to language bias, no updates on primary studies not included in published meta-analyses are planned, and that potential small-study effects due to inclusion of only peer-reviewed journal.

## Supplementary Information

**Additional file 1.** Search strategy in each database.

**Additional file 2.** Data extraction sheet.

**Additional file 3.** Methodology quality assessment checklist.

**Additional file 4.** Evidence strength assessment checklist.

**Additional file 5.** Pieper’s method of reporting overlap between systematic reviews.

**Additional file 6.** Munder’s method [1] for statistically managing overlap between meta-analyses.

**Additional file 7.** Formula for effect size transformation.

## Data Availability

Not applicable.

## Abbreviations

ALDH2: Aldehyde dehydrogenase type 2
4-HNE: 4-hydroxy-2-nonenal
MDA: Malondialdehyde
ALDH2*2: Aldehyde dehydrogenase type 2 deficient allele
Glu504Lys: Aldehyde dehydrogenase type 2 deficient allele
SNP: Single nucleotide polymorphism
rs671 G>A: a G to a mutation at codon 504 in exon 12 of aldehyde dehydrogenase type 2 gene located on chromosome 12q24
Glu: Glutamate
Lys: Lysine
PRISMA: Preferred Reporting Items for Systematic Review and Meta-analysis
PRISMA-P: Preferred Reporting Items for Systematic Reviews and Meta-analysis Protocols
PROSPERO: International Prospective Register of Systematic Reviews
HWE: Hardy-Weinberg Equilibrium
ES: Effect size
CI: Confidence interval
SE: Standard error
AMSTAR: The Measurement Tool to Assess the Methodological Quality of Systematic Reviews
AMSTAR2: a Critical Appraisal Tool for Systematic Reviews that Include Randomized or Non-randomized Studies of Healthcare Interventions, or Both
NOS: Newcastle-Ottawa Scale
STREGA: STrengthening the REporting of Genetic Association studies
GRADE: Grading of Recommendations Assessment, Development and Evaluation
CA: Covered area
CCA: Corrected covered area
Log_OR: Log odds ratio
