# Supplementary material for: Aldehyde dehydrogenase 2 rs671 polymorphism and multiple diseases: protocol for a quantitative umbrella review of meta-analyses
Source: Syst Rev. 2022 Sep 2;11:185. doi: 10.1186/s13643-022-02050-y (PMC9438126; doi:10.1186/s13643-022-02050-y)
Supplement: Supplementary file 1 — Additional file 1. Search strategy in each database. [file 13643_2022_2050_MOESM1_ESM.docx]

**Additional file 1. Search strategy in each database**

| **Database** | **Search strategy** |
| --- | --- |
| PubMed | ("Aldehyde Dehydrogenase, Mitochondrial/deficiency"[Mesh] OR (("ALDH2 protein, human" [Supplementary Concept] OR (ALDH2[Title/Abstract] OR ALDH-2[Title/Abstract] OR "ALDH 2"[Title/Abstract] OR "aldehyde dehydrogenase type 2"[Title/Abstract] OR "aldehyde dehydrogenase 2"[Title/Abstract] OR "aldehyde dehydrogenase-2"[Title/Abstract] OR "aldehyde dehydrogenase II"[Title/Abstract] OR "mitochondrial aldehyde dehydrogenase"[Title/Abstract] OR ALDM[Title/Abstract] OR "alcohol metabolism"[Title/Abstract]) AND  (("Genetic Variation"[Mesh] OR "Genotype"[Mesh] OR "Alleles"[Mesh]) OR (polymorphism*[Title/Abstract] OR variation*[Title/Abstract] OR variant*[Title/Abstract] OR mutation*[Title/Abstract] OR genotype*[Title/Abstract] OR allele*[Title/Abstract] OR SNP[Title/Abstract] OR rs671[Title/Abstract] OR G487A[Title/Abstract] OR 487Lys[Title/Abstract] OR 504Lys[Title/Abstract] OR "ALDH2*2"[Title/Abstract])))) AND  (((“systematic review*” OR “state-of-the-art review*” OR “scoping review*” OR “literature review*” OR “umbrella review*” OR “systematic overview*” OR “state-of-the-art overview*” OR “scoping overview*” OR “literature overview*” OR “umbrella overview*” OR “systematic assessment*” OR “state-of-the-art assessment*” OR “scoping assessment*” OR “literature assessment*” OR “umbrella assessment*”) OR "review* of reviews" OR meta-analy* OR metaanaly* OR ((systematic OR evidence) AND assess*) OR "research evidence" OR metasynthe* OR meta-synthe*)[Text Word] OR "Systematic Review" [Publication Type] OR "Systematic Reviews as Topic"[Mesh] OR "Meta-Analysis" [Publication Type] OR "Meta-Analysis as Topic"[Mesh]) |
| Medline (Ovid interface) | -1 exp “Aldehyde Dehydrogenase”/df [Deficiency]  -2 ALDH2.ab,ti. or ALDH-2.ab,ti. or “ALDH 2”.ab,ti. or “aldehyde dehydrogenase type 2”.ab,ti. or “aldehyde dehydrogenase 2”.ab,ti. or "aldehyde dehydrogenase-2".ab,ti. or “aldehyde dehydrogenase II”.ab,ti. or “mitochondrial aldehyde dehydrogenase”.ab,ti. or “ALDM”.ab,ti. or “alcohol metabolism”.ab,ti.  -3 exp “Genetic Variation”/ or exp Genotype/ or exp Alleles/  -4 "polymorphism*".ab,ti. or "variation*".ab,ti. or "variant*".ab,ti. or "mutation*".ab,ti. or "genotype*".ab,ti. or "allele*".ab,ti. or SNP.ab,ti. or rs671.ab,ti. or G487A.ab,ti. or 487Lys.ab,ti. or 504Lys.ab,ti. or "ALDH2*2".ab,ti.  -5 (((systematic or state-of-the-art or scoping or literature or umbrella) adj (review* or overview* or assessment*)) or "review* of reviews" or meta-analy* or metaanaly* or ((systematic or evidence) adj1 assess*) or "research evidence" or metasynthe* or meta-synthe*).tw. or (exp "Systematic Review"/ or exp “Systematic Reviews as Topic”/ or exp Meta-Analysis/ or exp “Meta-Analysis as Topic”/)  -6 3 or 4  -7 2 and 6  -8 1 or 7  -9 5 and 8 |
| Embase | ('aldh2 gene'/exp OR 'aldh2 protein human'/exp OR 'aldh2 protein'/exp OR 'aldehyde dehydrogenase isoenzyme 2'/exp OR 'aldehyde dehydrogenase 2 gene'/exp OR 'alcohol metabolism'/exp OR aldh2:ti,ab OR 'aldh 2':ti,ab OR "aldehyde dehydrogenase type 2":ti,ab OR 'aldehyde dehydrogenase 2':ti,ab OR 'aldehyde dehydrogenase-2':ti,ab OR 'aldehyde dehydrogenase ii':ti,ab OR 'mitochondrial aldehyde dehydrogenase':ti,ab OR 'alcohol metabolism':ti,ab) AND  ('polymorphism'/exp OR 'genetic polymorphism'/exp OR 'variation'/exp OR 'genetic variation'/exp OR 'variant'/exp OR 'gene mutation'/exp OR 'genotype'/exp OR 'allele'/exp OR 'snp'/exp OR polymorphism*:ti,ab OR variation*:ti,ab OR variant*:ti,ab OR mutation*:ti,ab OR genotype*:ti,ab OR allele*:ti,ab OR snp:ti,ab OR rs671:ti,ab OR g487a:ti,ab OR 487lys:ti,ab OR 504lys:ti,ab OR 'aldh2*2':ti,ab) AND  ((systematic:ti,ab,tn OR 'state of the art':ti,ab,tn OR scoping:ti,ab,tn OR literature:ti,ab,tn OR umbrella:ti,ab,tn) AND adj:ti,ab,tn AND (review*:ti,ab,tn OR overview*:ti,ab,tn OR assessment*:ti,ab,tn) OR 'review* of reviews':ti,ab,tn OR 'meta analy*':ti,ab,tn OR metaanaly*:ti,ab,tn OR ((systematic:ti,ab,tn OR evidence:ti,ab,tn) AND adj1:ti,ab,tn AND assess*:ti,ab,tn) OR 'research evidence':ti,ab,tn OR metasynthe*:ti,ab,tn OR 'meta synthe*':ti,ab,tn OR 'systematic review'/exp OR 'systematic review topic'/exp OR 'systematic review (topic)'/exp OR 'meta analysis'/exp OR 'meta analysis topic'/exp OR 'meta analysis (topic)'/exp) |
| Cochrane Database of Systematic Review | -1 MeSH descriptor: [Aldehyde Dehydrogenase, Mitochondrial] explode all trees and with qualifier(s): [deficiency - DF]  -2 MeSH descriptor: [Aldehyde Dehydrogenase, Mitochondrial] explode all trees  -3 (ALDH2 OR ALDH-2 OR "ALDH 2" OR "aldehyde dehydrogenase type 2" OR "aldehyde dehydrogenase 2" OR "aldehyde dehydrogenase-2" OR "aldehyde dehydrogenase ii" OR "mitochondrial aldehyde dehydrogenase" OR "alcohol metabolism"):ti,ab,kw  -4 (MeSH descriptor: [Polymorphism, Genetic] explode all trees) OR (MeSH descriptor: [Genetic Variation] explode all trees) OR (MeSH descriptor: [Genotype] explode all trees)  -5 (polymorphism* OR variation* OR variant* OR mutation* OR genotype* OR allele* OR snp OR rs671 OR g487a OR 487lys OR 504lys OR '"aldh2*2"):ti,ab,kw  -6 2 or 3  -7 4 or 5  -8 6 and 7  -9 1 or 8 |
| Web of Science  (All Databases) | (TI=(ALDH2 OR ALDH-2 OR "ALDH 2" OR "aldehyde dehydrogenase 2" OR "aldehyde dehydrogenase-2" OR "aldehyde dehydrogenase II" OR "mitochondrial aldehyde dehydrogenase" OR ALDM OR "alcohol consumption" OR "alcohol drinking" OR "alcohol intake" OR "alcohol metabolism") OR AB=(ALDH2 OR ALDH-2 OR "ALDH 2" OR "aldehyde dehydrogenase type 2" OR "aldehyde dehydrogenase 2" OR "aldehyde dehydrogenase-2" OR "aldehyde dehydrogenase II" OR "mitochondrial aldehyde dehydrogenase" OR ALDM OR "alcohol consumption" OR "alcohol drinking" OR "alcohol intake" OR "alcohol metabolism")) AND  (TI=(polymorphism* OR variation* OR variant* OR mutation* OR genotype* OR allele* OR SNP OR rs671 OR G487A OR 487Lys OR 504Lys OR "ALDH2*2") OR AB=(polymorphism* OR variation* OR variant* OR mutation* OR genotype* OR allele* OR SNP OR rs671 OR G487A OR 487Lys OR 504Lys OR "ALDH2*2")) AND  (TI=(((systematic OR state-of-the-art OR scoping OR literature OR umbrella) near/0 (review* OR overview* OR assessment*) ) OR "review* of reviews" OR meta-analy* OR metaanaly* OR ((systematic OR evidence) near/1 assess*) OR "research evidence" OR metasynthe* OR meta-synthe*) OR AB=(((systematic OR state-of-the-art OR scoping OR literature OR umbrella) near/0 (review* OR overview* OR assessment*) ) OR "review* of reviews" OR meta-analy* OR metaanaly* OR ((systematic OR evidence) near/1 assess*) OR "research evidence" OR metasynthe* OR meta-synthe*)) |
