# Supplementary material for: Aldehyde dehydrogenase 2 rs671 polymorphism and multiple diseases: protocol for a quantitative umbrella review of meta-analyses
Source: Syst Rev. 2022 Sep 2;11:185. doi: 10.1186/s13643-022-02050-y (PMC9438126; doi:10.1186/s13643-022-02050-y)
Supplement: Supplementary file 2 — Additional file 2. Data extraction sheet. [file 13643_2022_2050_MOESM2_ESM.docx]

**Additional file 2. Data extraction sheet**

**2-1 Data extraction sheet (meta-analyses level)**

| **First author** | **Year** | **Exposure** | **Comparison** | **Outcome** | **N** | **k** | **Study design of PRs** | **Quality assessment tool for PRs** | **Pooled**  **Effect size** | **CI/SE** | **Measurement of effect size** | **p-value of pooled effect size** | **Genetic model** | **I^2^** | **Comments** |
| --- | --- | --- | --- | --- | --- | --- | --- | --- | --- | --- | --- | --- | --- | --- | --- |
|  |  |  |  |  |  |  |  |  |  |  |  |  |  |  |  |
|  |  |  |  |  |  |  |  |  |  |  |  |  |  |  |  |
|  |  |  |  |  |  |  |  |  |  |  |  |  |  |  |  |
|  |  |  |  |  |  |  |  |  |  |  |  |  |  |  |  |
|  |  |  |  |  |  |  |  |  |  |  |  |  |  |  |  |

N: total number of participants; k: number of primary studies; PRs: primary studies; CI: confidential interval; SE: standard error

Content of “Exposure”: ALDH2*2 or ALDH2*1, indicating whether the exposure is mutation type (A) or wide type (G)

Content of “Comparison”: ALDH2*1 or ALDH2*2, indicating whether the comparison is wide type (G) or mutation type (A)

Content of “Genetic model”: allelic, dominant, heterozygous, homozygous, or recessive

**2-2 Data extraction sheet (primary studies level)**

| **Meta-analysis**  **(First author)** | **Meta-analysis**  **(Year)** | **Primary study**  **(First author)** | **Primary study**  **(Year)** | **Country (Ethnicity)** | **Study design** | **Source of control** | **Alcohol consumption** | **Gender** | **Exposure** | **Comparison** | **Precalculated Effect size** | **CI/SE** | **Measurement of effect size** | **Genetic model** | **HWE p-value** | **Comments** |
| --- | --- | --- | --- | --- | --- | --- | --- | --- | --- | --- | --- | --- | --- | --- | --- | --- |
|  |  |  |  |  |  |  |  |  |  |  |  |  |  |  |  |  |
|  |  |  |  |  |  |  |  |  |  |  |  |  |  |  |  |  |
|  |  |  |  |  |  |  |  |  |  |  |  |  |  |  |  |  |
|  |  |  |  |  |  |  |  |  |  |  |  |  |  |  |  |  |
|  |  |  |  |  |  |  |  |  |  |  |  |  |  |  |  |  |

CI: confidential interval; SE: standard error

Content of “Exposure”: ALDH2*2 or ALDH2*1, indicating whether the exposure is mutation type (A) or wide type (G)

Content of “Comparison”: ALDH2*1 or ALDH2*2, indicating whether the comparison is wide type (G) or mutation type (A)

Content of “Genetic model”: allelic, dominant, heterozygous, homozygous, or recessive

**2-3 Data extraction sheet (resolve conflicts of precalculated effect size in multiple meta-analyses)**

| **Meta-analysis**  **(First author)** | **Meta-analysis**  **(Year)** | **Primary study**  **(First author)** | **Primary study**  **(Year)** | **Country (Ethnicity)** | **Study design** | **Source of control** | **Alcohol consumption** | **Gender** | **Exposure** | **Comparison** | **Case group:**  **GG** | **Case group:**  **AG** | **Case group:**  **AA** | **Control group:**  **GG** | **Control group:**  **AG** | **Control group:**  **AA** | **Comments** |
| --- | --- | --- | --- | --- | --- | --- | --- | --- | --- | --- | --- | --- | --- | --- | --- | --- | --- |
|  |  |  |  |  |  |  |  |  |  |  |  |  |  |  |  |  |  |
|  |  |  |  |  |  |  |  |  |  |  |  |  |  |  |  |  |  |
|  |  |  |  |  |  |  |  |  |  |  |  |  |  |  |  |  |  |
|  |  |  |  |  |  |  |  |  |  |  |  |  |  |  |  |  |  |
|  |  |  |  |  |  |  |  |  |  |  |  |  |  |  |  |  |  |
|  |  |  |  |  |  |  |  |  |  |  |  |  |  |  |  |  |  |

Content of “Exposure”: ALDH2*2 or ALDH2*1, indicating whether the exposure is mutation type (A) or wide type (G)

Content of “Comparison”: ALDH2*1 or ALDH2*2, indicating whether the comparison is wide type (G) or mutation type (A)

GG: wide-type homozygous, Glu/Glu; AG: heterozygous, Glu/Lys; AA: mutation type homozygous, Lys/Lys
