# Supplementary material for: Aldehyde dehydrogenase 2 rs671 polymorphism and multiple diseases: protocol for a quantitative umbrella review of meta-analyses
Source: Syst Rev. 2022 Sep 2;11:185. doi: 10.1186/s13643-022-02050-y (PMC9438126; doi:10.1186/s13643-022-02050-y)
Supplement: Supplementary file 3 — Additional file 3. Methodology quality assessment checklist. [file 13643_2022_2050_MOESM3_ESM.docx]

**Additional file 3. Methodology quality assessment checklist**

**3-1 AMSTAR critical appraisal tool with specification and modification**

| **AMSTAR2 questions/criteria** | **Dichotomous questions used to assess quality of reviews** | **Answer** |
| --- | --- | --- |
| 1. Did the research questions and inclusion criteria for the review include the components of PECO? | *For Partial Yes, ALL of the following:*  1.1 The research question includes the components of PECO  1.2 The inclusion criteria for the review includes the components of PECO | Yes  Partial Yes  No |
|  | *For Yes, plus ALL of the following:*  1.3 The inclusion criteria for the review is clear for the components of PECOS:  Outcome: a clear diagnostic criteria for the outcome of interests  Study design: a clear statement of the study type included  1.4 (Optional) Timeframe for follow-up |  |
| 2. * Did the report of the review contain an explicit statement that the review methods were established prior to the conduct of the review and did the report justify any significant deviations from the protocol? | *For Partial Yes:*  2.1 The authors state that they had a written protocol or guide that included ALL the following:  Review question(s)  A search strategy  Inclusion/exclusion criteria  A risk of bias assessment | Yes  Partial Yes  No |
|  | *For Yes, plus ALL of the following:*  2.2 the protocol should also be registered  2.3 Registration record should also have specified:  A meta-analysis/synthesis plan  A plan for investigating causes of heterogeneity  Justification for any deviations from the protocol |  |
| 3. Did the review authors explain their selection of the study designs for inclusion in the review? | *Since the exposure of interests is a single nucleotide polymorphism, it is not feasible nor ethical to conduct randomized controlled trials. As for observational studies, through preliminary searching, we noticed that majority of primary studies on this topic is case-control study design, with a few cohort studies or cross-sectional studies. Considering the strength of evidence provided by cross-sectional studies, most studies included case-control and cohort studies. Since we consider the reason as a common sense, the answer for this question of all included reviews will be “Not Applicable”.* | N/A |
| 4. * Did the review authors use a comprehensive literature search strategy? | *For Partial Yes, ALL of the following:*  Searched at least 2 databases (relevant to research question)  Provide key word and/or search strategy  Justified publication restrictions (eg, language) | Yes  Partial Yes  No |
|  | *For Yes, plus ALL of the following:*  Search the reference lists/bibliographies of included studies  Included/consulted content experts in the field  Where relevant, searched for grey literature  Conducted search within 24 months of completion of the review |  |
| 5. Did the review authors perform study selection in duplicate? | *For Yes, either ONE of the following:*  At least two reviewers independently agreed on selection of eligible studies and achieved consensus on which studies to include  Two reviewers selected a sample of eligible studies and achieved good agreement (at least 80 per cent), with the remainder selected by one reviewer | Yes  No |
| 6. Did the review authors perform data extraction in duplicate? | *For Yes, either ONE of the following:*  At least two reviews achieved consensus on which data to extract from included studies  Two reviewers extracted data from a sample of eligible studies and achieved good agreement (at least 80 per cent), with the remainder extracted by one reviewer | Yes  No |
| 7. * Did the review authors provide a list of excluded studies and justify the exclusions? | *For Partial Yes:*  7.1 Provided a list of all potentially relevant studies that were read in full text form but excluded from the review | Yes  Partial Yes  No |
|  | *For Yes, must also have:*  7.2 Justified the exclusion from the review of each potentially relevant study |  |
| 8. Did the review authors describe the included studies in adequate detail? | *For Partial Yes, ALL of the following:*  Described populations  Described exposures  Described comparators  Described outcomes  Described research designs | Yes  Partial Yes  No |
|  | *For Yes, plus ALL of the following:*  Described population in detail: gender, ethnic or country must be described  Described exposure and comparator in detail: mutant as exposure and wide-type as comparator or vice versa must be described  Described study design in detail: whether cohort studies are prospective or retrospective, whether case-control studies are hospital-based or population-based, must be described |  |
| 9. * Did the review authors use a satisfactory technique for assessing the risk of bias (RoB) in individual studies that were included in the review? | *For Partial Yes, ALL of the following:*  9.1 RoB (or methodologic quality) formally assessed:  All of the following:  The scientific quality of included studies was assessed  The assessment was done by at least 2 independent reviews  The author stated the tool used for quality assessment  The scientific quality of studies was documented  9.2 Components of the assessment stated by the author or in the tool used by the author included ALL of the following:  Selection of participants  Confounding | Yes  Partial Yes  No |
|  | *For Yes, plus:*  9.3 The assessment included ALL of the following:  Criteria to ascertain all exposures and outcomes  selection of the reported result from among multiple measurements or analyses of a specified outcome  9.4 (Optional) The 4 criteria listed above need to be documented separately for each study |  |
| 10. Did the review authors report on the sources of funding for the studies included in the review? | *For Yes:*  Must have reported on the sources of funding for individual studies included in the review. Note: Reporting that the reviewers looked for this information but it was not reported by study authors also qualifies | Yes  No |
| 11. * Did the review authors use appropriate methods for statistical combination of results in meta-analysis? | *For Partial Ye:*  The author used an appropriate weighted technique to combine study results, adjusting for heterogeneity if present | Yes  Partial Yes  No |
|  | *For Yes, either ONE of the following:*  The author statistically combined effect estimates from observational studies that were adjusted for confounding, rather than combining raw data, or justified combining raw data when adjusted effect estimates were not available  The author separated raw data as subgroup according to confounding factors, and conducted subgroup analysis to address confounding |  |
| 12. Did the review authors assess the potential impact of RoB in individual studies on the results of the meta-analysis or other evidence synthesis? | *For Yes, either ONE of the following:*  The author stated that quality of all included studies are acceptable  Included only low risk of bias studies  If the pooled estimate was based on studies at variable RoB, the authors performed analyses to investigate possible impact of RoB on summary estimates of effect | Yes  No |
| 13. * Did the review authors account for RoB in individual studies when interpreting/discussing the results of the review? | *For Yes, either ONE of the following:*  The author stated that quality of all included studies are acceptable  Included only low risk of bias studies  If studies with moderate or high RoB were included the review provided a discussion of the likely impact of RoB on the results | Yes  No |
| 14. Did the review authors provide a satisfactory explanation for, and discussion of, any heterogeneity observed in the results of the review? | *For Yes, ALL of the following:*  14.1 Heterogeneity was presented  14.2 Either ONE of the following:  Heterogeneity was interpreted as not significant in the results  The authors performed an investigation of sources of any heterogeneity in the results and discussed the impact of this on the results of the review | Yes  No |
| 15. * Did the review authors carry out an adequate investigation of publication bias (small study bias) and discuss its likely impact on the results of the review? | *For Yes, ALL of the following*  15.1 Publication bias were presented by either ONE of the following:  Graphical presentation (eg. funnel plot)  Statistical tests  15.2 The likelihood and magnitude of impact of publication bias were discussed | Yes  No |
| 16. Did the review authors report any potential sources of conflict of interest, including any funding they received for conducting the review? | *For Yes, either ONE of the following:*  The authors reported no competing interests  The authors described their funding sources and how they managed potential conflicts of interest | Yes  No |

* indicates critical items

**3-2 Rating overall quality in the results of the review**

| **Quality** | **Criteria** |
| --- | --- |
| High | *No or one non-critical weakness*: the systematic review provides an accurate and comprehensive summary of the results of the available studies that address the question of interest |
| Moderate | *More than one non-critical weakness**: the systematic review has more than one weakness but no critical flaws. It may provide an accurate summary of the results of the available studies that were included in the review |
| Low | *One critical flaw with or without non-critical weaknesses:* the review has a critical flaw and may not provide an accurate and comprehensive summary of the available studies that address the question of interest |
| Critically low | *More than one critical flaw with or without non-critical weaknesses:* the review has more than one critical flaw and should not be relied on to provide an accurate and comprehensive summary of the available studies |

*Multiple non-critical weaknesses may diminish confidence in the review and it may be appropriate to move the overall appraisal down from moderate to low confidence

**3-3 Methodology quality assessment table**

| **Author, year** | **AMSTAR2 items** | | | | | | | | | | | | | | | | **Number** | | | |
| --- | --- | --- | --- | --- | --- | --- | --- | --- | --- | --- | --- | --- | --- | --- | --- | --- | --- | --- | --- | --- |
|  | **1** | **2*** | **3** | **4*** | **5** | **6** | **7*** | **8** | **9*** | **10** | **11*** | **12** | **13*** | **14** | **15*** | **16** | **Y** | **P** | **N** | **N/A** |
|  |  |  |  |  |  |  |  |  |  |  |  |  |  |  |  |  |  |  |  |  |
|  |  |  |  |  |  |  |  |  |  |  |  |  |  |  |  |  |  |  |  |  |
|  |  |  |  |  |  |  |  |  |  |  |  |  |  |  |  |  |  |  |  |  |
|  |  |  |  |  |  |  |  |  |  |  |  |  |  |  |  |  |  |  |  |  |
|  |  |  |  |  |  |  |  |  |  |  |  |  |  |  |  |  |  |  |  |  |
|  |  |  |  |  |  |  |  |  |  |  |  |  |  |  |  |  |  |  |  |  |
|  |  |  |  |  |  |  |  |  |  |  |  |  |  |  |  |  |  |  |  |  |
|  |  |  |  |  |  |  |  |  |  |  |  |  |  |  |  |  |  |  |  |  |
|  |  |  |  |  |  |  |  |  |  |  |  |  |  |  |  |  |  |  |  |  |
|  |  |  |  |  |  |  |  |  |  |  |  |  |  |  |  |  |  |  |  |  |
|  |  |  |  |  |  |  |  |  |  |  |  |  |  |  |  |  |  |  |  |  |
|  |  |  |  |  |  |  |  |  |  |  |  |  |  |  |  |  |  |  |  |  |
|  |  |  |  |  |  |  |  |  |  |  |  |  |  |  |  |  |  |  |  |  |
|  |  |  |  |  |  |  |  |  |  |  |  |  |  |  |  |  |  |  |  |  |
|  |  |  |  |  |  |  |  |  |  |  |  |  |  |  |  |  |  |  |  |  |

Y: yes; P: partial yes; N: no; N/A: unapplicable; * indicates critical items
