# Supplementary material for: Aldehyde dehydrogenase 2 rs671 polymorphism and multiple diseases: protocol for a quantitative umbrella review of meta-analyses
Source: Syst Rev. 2022 Sep 2;11:185. doi: 10.1186/s13643-022-02050-y (PMC9438126; doi:10.1186/s13643-022-02050-y)
Supplement: Supplementary file 4 — Additional file 4. Evidence strength assessment checklist. [file 13643_2022_2050_MOESM4_ESM.docx]

**Additional file 4. Evidence strength assessment checklist**

| **Level of evidence strength** | **Criteria** |
| --- | --- |
| convincing evidence  (class I) | >1000 cases  significant summary associations (P<10^−6^) per random‐effects calculation  no evidence of small‐study effects  no evidence of excess of significance bias  prediction intervals not including the null  largest study nominally significant (p < 0.05)  heterogeneity not large (I^2^<50%) |
| highly suggestive evidence  (class II) | significant summary associations (*P*<10^−6^) per random‐effects calculation  >1000 cases  the largest study with 95% CI excluding the null |
| suggestive evidence  (class III) | >1000 cases  significant summary associations (*P*<10^−3^) per random‐effects calculation |
| weak evidence  (class IV) | all other risk factors with *P*<0.05 |
| non‐significant associations  (class V) | all associations with *P*>0.05 |

note: evidence strength will be assessed under each genetic model and each outcome for each included meta-analysis
