# Supplementary material for: Aldehyde dehydrogenase 2 rs671 polymorphism and multiple diseases: protocol for a quantitative umbrella review of meta-analyses
Source: Syst Rev. 2022 Sep 2;11:185. doi: 10.1186/s13643-022-02050-y (PMC9438126; doi:10.1186/s13643-022-02050-y)
Supplement: Supplementary file 5 — Additional file 5. Pieper’s method of reporting overlap between systematic reviews. [file 13643_2022_2050_MOESM5_ESM.docx]

**Additional file 5. Pieper’s method of reporting overlap between systematic reviews**

**5-1 Format of citation matrix[1]**

**Primary study 2 is included in systematic review 1 and systematic review r but not in systematic review 2**

|  | **Systematic review 1** | **Systematic review 2** | **……** | **Systematic review r** |
| --- | --- | --- | --- | --- |
| **Primary study 1** |  | x |  |  |
| **Primary study 2** | x |  |  | x |
| **……** |  |  |  |  |
| **Primary study c** | x | x |  | x |

**5-2 Formula for covered area (CA) and corrected covered area (CCA)[1]**

$$CA= \frac{N}{r c}$$

$$CCA= \frac{N-r}{r c-r}$$

*N*: the number of included publications (including double counting) in evidence synthesis (this is the sum of the ticked boxes in the citation matrix)

*r*: the number of rows (number of primary studies)

*c*: the number of columns (number of systematic reviews)

Interpretation of CCA[1]:

| CCA | Overlap |
| --- | --- |
| 0 – 5 | Slight |
| 6 – 10 | Moderate |
| 11 - 15 | High |
| > 15 | Very high |

**References:**

1. Pieper D, Antoine S-L, Mathes T, Neugebauer EAM, Eikermann M. Systematic review finds overlapping reviews were not mentioned in every other overview. Journal of Clinical Epidemiology. 2014;67(4):368-75.
