# Supplementary material for: Aldehyde dehydrogenase 2 rs671 polymorphism and multiple diseases: protocol for a quantitative umbrella review of meta-analyses
Source: Syst Rev. 2022 Sep 2;11:185. doi: 10.1186/s13643-022-02050-y (PMC9438126; doi:10.1186/s13643-022-02050-y)
Supplement: Supplementary file 6 — Additional file 6. Munder’s method [1] for statistically managing overlap between meta-analyses. [file 13643_2022_2050_MOESM6_ESM.docx]

**Additional file 6. Munder’s method[1] for statistically managing overlap between meta-analyses**

**6-1 Formula for managing overlap of Munder et al. 2013[1]**

**1) Transformation of r into Fisher’s Z:**

$$Z=0.5\ln\left[ \frac{1+r}{1-r} \right]$$

which follows normal distribution $N(\frac{1}{2}\ln\left( \frac{1+\rho}{1 -\rho} \right), \frac{1}{\left( N-3 \right)^{\frac{1}{2}}})$

r: Pearson correlation coefficient; ρ: true correlation coefficient; N: sample size

**2) Calculating uniqueness (U) of each primary study i:**

$$U_{i}= \frac{1}{n}$$

n: the number of meta-analyses in which primary study I was included

**3) Calculating adjusted number of primary studies (k_adj_) for each meta-analysis j:**

$$k_{adj, j}= \sum U_{i}$$

**4) Calculating the standard error for the effect from meta-analysis j (SE_j_):**

$${SE}_{j}= \frac{1}{{(k_{adj, j}-3)}^{\frac{1}{2}}}$$

**5) Calculating overlap-corrected weight (W) for each meta-analysis j:**

$$W_{j}= \frac{1}{\tau^{2}+ {{SE}_{j}}^{2}}$$

N: sample size; r: Pearson correlation coefficient

**6-2 Explanation**

Munder et al. 2013’s method was originated from a method to manage overlapping populations in primary studies.[1] For each primary study i, the number of meta-analyses included this primary study (n_i_) was counted. The inverse of n_i_ (U_i_) was regarded as the uniqueness of this study. For each meta-analysis j, the overlap-adjusted number of primary studies (k_adj,j_) was calculated as the sum of U_i_ of all included primary studies. Pooled effect size of all meta-analyses was transformed to Fisher Z. Standard error of the effect size for each meta-analysis j (SE_j_) was calculated using Fisher Z’s standard error formula and k_adj,j_ was used as the sample size during the calculation. The overlap-adjusted weight for each meta-analysis j was calculated based on variation of the true effects τ^2^ and SE_j_.

This method is further adopted by 2 other meta-meta-analyses[2, 3]. However, the methodology of these 2 studies is in doubt, since they transformed pooled effect size to standard mean deviation (SMD), however, SE_j_ was still calculated using Fisher Z’s standard error formula.

Standard error of standard mean difference (SMD) [4]:

$$SE\left( SMD \right)= \sqrt{\frac{1}{N}+ \frac{{SMD}^{2}}{2N}} \times\sqrt{2 (1-r)}$$

MD: mean difference**References:**

1. Munder T, Bruetsch O, Leonhart R, Gerger H, Barth J. Researcher allegiance in psychotherapy outcome research: an overview of reviews. Clinical Psychology Review. 2013;33(4):501-11.

2. Weber L, Kamp-Becker I, Christiansen H, Mingebach T. Treatment of child externalizing behavior problems: A comprehensive review and meta–meta-analysis on effects of parent-based interventions on parental characteristics. European Child & Adolescent Psychiatry. 2019;28(8):1025-36.

3. Mingebach T, Kamp-Becker I, Christiansen H, Weber L. Meta-meta-analysis on the effectiveness of parent-based interventions for the treatment of child externalizing behavior problems. PloS one. 2018;13(9):e0202855.

4. Higgins JPT GSe. Cochrane Handbook for Systematic Reviews of Interventions Version 5.1.0 [updated March 2011]. The Cochrane Collaboration. 2011.
