# Supplementary material for: Aldehyde dehydrogenase 2 rs671 polymorphism and multiple diseases: protocol for a quantitative umbrella review of meta-analyses
Source: Syst Rev. 2022 Sep 2;11:185. doi: 10.1186/s13643-022-02050-y (PMC9438126; doi:10.1186/s13643-022-02050-y)
Supplement: Supplementary file 7 — Additional file 7. Formula for effect size transformation. [file 13643_2022_2050_MOESM7_ESM.docx]

**Additional file 7. Formula for effect size transformation**

Formula for transforming effect size to Log Odds Ratio and Standard Error of Log Odds Ratio

$$log(OR)=\ln\left( OR \right)=ln(\frac{ad}{bc})$$

$$CI\left( \log\left( OR \right) \right)=\ln\left( CI\left( OR \right) \right)$$

$$SE\left( \log\left( OR \right) \right)= \frac{1}{3.92}\times({CI\left( \log\left( OR \right) \right)}_{upper}-{CI(\log\left( OR \right))}_{lower})= \sqrt{\frac{1}{a}+\frac{1}{b}+\frac{1}{c}+\frac{1}{d}}$$

OR: odds ratio; SE: standard error; a: number of exposed cases; b : number of exposed non-cases; c: number of unexposed cases; d: number of unexposed ono-cases
